# Bloch Surface Waves Biosensors for High Sensitivity Detection of Soluble ERBB2 in a Complex Biological Environment

**DOI:** 10.3390/bios7030033

**Published:** 2017-08-17

**Authors:** Alberto Sinibaldi, Camilla Sampaoli, Norbert Danz, Peter Munzert, Frank Sonntag, Fabio Centola, Agostino Occhicone, Elisa Tremante, Patrizio Giacomini, Francesco Michelotti

**Affiliations:** 1 Department of Basic and Applied Science for Engineering, Sapienza University of Rome, Via A. Scarpa 16, 00161 Rome, Italy; agostino.occhicone@uniroma1.it (A.O.); francesco.michelotti@uniroma1.it (F.M.); 2 Regina Elena National Cancer Institute, Via E. Chianesi 53, 00144 Rome, Italy; camilla.sampaoli@gmail.com (C.S.); etremante@gmail.com (E.T.); patrizio.giacomini@ifo.gov.it (P.G.); 3 Fraunhofer Institute for Applied Optics and Precision Engineering IOF, Albert-Einstein-Str. 7, 07745 Jena, Germany; norbert.danz@iof.fraunhofer.de (N.D.); peter.munzert@iof.fraunhofer.de (P.M.); 4 Fraunhofer Institute for Material and Beam Technology IWS, Winterbergstr. 28, 01277 Dresden, Germany; frank.sonntag@iws.fraunhofer.de; 5 IBI-Istituto Biochimico Italiano Giovanni Lorenzini Spa, Via Fossignano 2, 04011 Aprilia, Italy; fcentola@ibi-lorenzini.com

**Keywords:** Optical biosensors, Bloch surface waves, 1D photonic crystals, ERBB2, SK-BR 3, Breast cancer

## Abstract

We report on the use of one-dimensional photonic crystals to detect clinically relevant concentrations of the cancer biomarker ERBB2 in cell lysates. Overexpression of the ERBB2 protein is associated with aggressive breast cancer subtypes. To detect soluble ERBB2, we developed an optical set-up which operates in both label-free and fluorescence modes. The detection approach makes use of a sandwich assay, in which the one-dimensional photonic crystals sustaining Bloch surface waves are modified with monoclonal antibodies, in order to guarantee high specificity during the biological recognition. We present the results of exemplary protein G based label-free assays in complex biological matrices, reaching an estimated limit of detection of 0.5 ng/mL. On-chip and chip-to-chip variability of the results is addressed too, providing repeatability rates. Moreover, results on fluorescence operation demonstrate the capability to perform high sensitive cancer biomarker assays reaching a resolution of 0.6 ng/mL, without protein G assistance. The resolution obtained in both modes meets international guidelines and recommendations (15 ng/mL) for ERBB2 quantification assays, providing an alternative tool to phenotype and diagnose molecular cancer subtypes.

## 1. Introduction

Over-expression of the human erb-b2 receptor tyrosine kinase 2 (ERBB2) marks the acquisition of growth factor independence by primary and metastatic cancers, and dictates therapeutic ERBB2 blockade by small drugs and antibodies [1]. In the absence of appropriate therapy, altered ERBB2 expression (occurring in about 20–25% of invasive breast cancers) is associated with poor-prognosis and a decrease of survival chances [2]. The soluble cleaved form of the ERBB2 protein (s-ERBB2), found in blood has been demonstrated to be a valuable marker for subtype assessment [3].

From a biochemical point of view, the ERBB2 glycoprotein (185 kDa) is a receptor tyrosine kinase belonging to the family of the epidermal growth factor receptors (EGFRs), involved in cell signaling during proliferation, growth and differentiation [4]. ERBB2 shows very low basal expression in many tissues types, and is involved in normal tissue development and function [4,5]. For this reason, it is clinically important to discriminate extra cellular domain (ECD) ERBB2 levels across a wide range of concentrations, from small to very high.

Nowadays, immuno-histochemical (IHC) staining techniques are widely used to obtain a semi-quantitative estimation of ERBB2 in tumor tissues. Fluorescence in situ hybridization (FISH) exploits labelled DNA probes to determine the Copy Number Variation (CNV) of the ERBB2 gene in Formalin-Fixed Paraffin-Embedded (FFPE) tissue sections. In 2000, the FDA approved and certified 15 ng/mL is the upper limit of ERBB2 in the serum of healthy subjects, thus pushing the medical community to use such a value as a reliable indicator of anti-tumor treatment efficiency. On the other hand, it has been shown that a high serum level of ECD ERBB2 can indicate resistance to immuno-therapeutic treatment such as Trastuzumab (Herceptin^®^, Roche, Basel, Switzerland) or Pertuzumab (Omnitarg^®^, Genentech, South San Francisco, CA, USA). Cell lysates are one of the most heterogeneous and difficult environments for biosensing applications due to their intrinsic complexity. Thus, reaching the 15 ng/mL threshold established in serum may be particularly challenging if ERBB2 has to be detected in cell lysates. Recently, new promising approaches making use of nanobodies [6], nanoelectrode arrays [7] and amperometric magneto-immunosensor [8] were described that guarantee limits of detection (LoD) comparable with ELISA ERBB2 kits (0.2 ng/mL). In particular, Eletziguerra et al. [8] measured ERBB2 in living cells directly, and discriminated differences between three different cell lines. 

In this work, the use of a real-time label-free and fluorescence based optical set-up has been pursued as a quantitative alternative to IHC and Western Blot (WB). The optical transducer is based on a one-dimensional photonic crystal (1DPC) that consists of a dielectric multilayer with suitable refractive index contrast and transparency, supporting Bloch surface waves (BSW) either in the near infrared [9,10] or in the visible spectral range [11,12]. The relevance of a combined label-free and fluorescence approach was previously demonstrated [13]. Here we aim to analyze in detail the two operation mechanisms in order to characterize independently the stability of label-free response and peculiar fluorescence features. An estimation of the LoD in label-free and fluorescence modes is obtained. A BSW can be excited by a prism coupler leading to a dip in the angular reflectance spectrum. The angular position of such a dip is very sensitive to any perturbation of the refractive index at the interface and is exploited for label-free bio-sensing. Besides this, fluorescent molecules at the 1DPC surface yield surface wave enhanced emission, which is utilized to obtain further information on the cancer biomarker assay. We report experimental results obtained in label-free and fluorescence operation modes for ERBB2 positive and negative lysates in contact with a BSW biochip. Such results permit a direct comparison of the biosensing performances of the BSW chips, addressing the limit of detection for ERBB2 in a complex biological matrix. Consequently, a limit of detection for ERBB2 positive cell lysates is provided for both label-free and fluorescence modes.

## 2. Materials and Methods

### 2.1. Cell Biology and Biochemistry 

For the present study, we used two different cell lines: SK-BR 3 and Colo 38. SK-BR-3 breast cancer cells carry an amplified and overexpressed ERBB2 gene, and were used as a convenient source of ERBB2 molecules. Colo 38 melanoma cells were selected as a control lysate since they do not express detectable amounts of ERBB2 [14]. The SK-BR 3 and Colo 38 cell lines were grown in RPMI 1640 medium containing bovine serum. Their identity was periodically verified by DNA genotyping [15]. Soluble cell lysates were prepared by incubating 1 × 10^7^ cells on ice for 30 min in 1 mL of D-PBS 1 X (pH 7.0) containing the non-ionic detergent Nonidet P40 (1% NP40) as well as antiproteases aprotinin at 1 μg/mL and phenyl-methyl-sulphonyl-fluoride (PMSF) at 1 mM, both from Sigma-Aldrich. The total protein concentration C_WH_ in the lysates was determined by a modified Bradford protein assay (BCA, Biorad).

**Figure 1:**
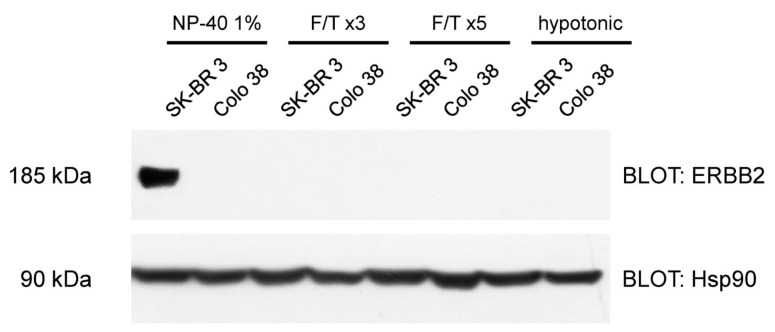
Western Blot of SK-BR 3 and Colo 38 lysates for different extraction strategies. A heat shock protein of 90 kDa was used as a control.

Figure 1 shows the WB of SK-BR 3 and Colo 38 lysates for different extraction recipes. It can be noted that the use of 1% NP40 guarantees the complete extraction of membrane proteins with respect to freeze-thaw (F/T) cycles and hypotonic solution based strategies. In particular, an increase in the F/T cycles from ×3 to ×5 does not improve the ERBB2 extraction efficiency. Nevertheless, as confirmed in Figure 1, these latter lysis systems permit the efficient extract of proteins from the cytosolic fraction, e.g., heat shock protein Hsp 90. The total lysate used in the WBs was 50 μg/lane [16].

We used the following approach to calculate the number of soluble ERBB2 molecules in our experimental samples. SK-BR 3 lysates were prepared at a known cell concentration (1.5 × 10^4^ cell/μL), and total proteins in lysates were assessed. Since a single SK-BR-3 cell expresses 5 × 10^5^ ERBB2 proteins [17,18], it is easy to estimate that, under the above solubilisation conditions, 1 μg of NP40 extract contains 7.5 × 10^9^ ERBB2 molecules.

The total number of ERBB2 proteins per cell is still under debate. Such uncertainty renders the estimated ERBB2 concentrations used in the present work inaccurate. However, the value 5 × 10^5^ ERBB2 receptors per cell in overexpressing ERBB2-amplified cells was confirmed by two independent and recent works [17,18], indicating a good accuracy. To increase the accuracy of the ERBB2 concentration estimation, one should carry out a calibration with known solutions of ERBB2 in a truly negative lysate, which is being performed but is not yet reported in this work.

Monoclonal antibodies (mAbs) W6/300G9 (capture Anti-ERBB2) and W6/800E6 (detection Anti-ERBB2) bind distinct epitopes of the ERBB2 ectodomain [19]. For fluorescence detection, mAb W6/800E6 was conjugated to the NHS ester of Alexa Fluor 647 at an approximate molar ratio of 10:1 (Anti-ERBB2 AF647). For internal background referencing mAbs of irrelevant specificity were selected. mAb L31 binds human major histocompatibility complex class I (MHC I) molecules [20]. All mAbs were dissolved in Dulbecco’s phosphate buffered saline 1 X (D-PBS 1 X). Sulfuric acid (95—98%), hydrogen peroxide (30% in H_2_O), (3-aminopropyl)triethoxysilane (APTES, 99%), ethanol (99.8%), glutaraldehyde solution (grade I, 50% in H_2_O), sodium bicarbonate (99.7%), sodium cyanoborohydride (95%), hydrogen chloride (2M), glycine (99%), bovine serum albumin (BSA, 98%), and D-PBS 10 X (100 mM) were purchased from Sigma-Aldrich. Alexa Fluor 647 NHS ester (1 mg/mL) and protein G (PtG) were purchased from Thermo Fisher Scientific.

### 2.2. BSW Chips

**Figure 2:**
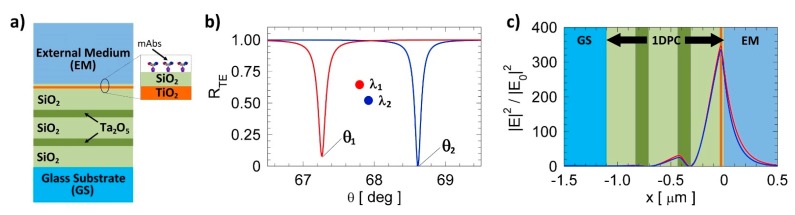
(**a**) Sketch of the 1DPC geometry; (**b**) Numerically calculated TE intensity reflectance vs. angle for the 1DPC sketched in Figure 2a in water at λ_1_ (red curve) and λ_2_ (blue curve), respectively; (**c**) Numerically calculated distribution of |E|^2^ of the TE-polarized BSW at λ_1_ and λ_2_ at corresponding resonance angles (θ_1_ and θ_2_).

One dimensional photonic crystals are layered optical media constituted by artificial structures where a periodic modulation of the permittivity occurs in one direction only (Figure 2a). Their most important property is the presence of the photonic band gap (PBG) that refers to a wavelength range where light propagation is prohibited inside the 1DPC. BSW lie in such forbidden bands and are localized at the interface between a finite 1DPC and an external medium by Bragg reflection and total internal reflection (TIR) on the two sides of the interface, respectively [21]. The excitation of a BSW at a given wavelength can be obtained by a prism coupler in the so-called Kretschmann-Raether configuration [22] and is revealed by the appearance of a dip in the angular reflectance, as shown in the curves shown in Figure 2b, which were calculated for a water environment and for the TE polarization. Due to the BSW dispersion it is possible to tune the resonance angle by just changing the wavelength. The simulations for the two illumination wavelengths used in the label-free (λ_1_ = 670 nm, red curve) and fluorescence (λ_2_ = 635 nm, blue curve) mode were done by means of the transfer matrix method. Figure 2c depicts the transverse|E|^2^ field distribution at resonance for both TE polarized BSW at λ_1_ and λ_2_.

The label-free mode works at λ_1_. Similarly to other surface wave based biosensors, sensing relies on tracking the resonance position (θ_1_) during the bio-recognition assay. The change of the resonance position can be associated with mass loading at the 1DPC surface and converted to a mass surface coverage (ng/cm^2^) revealing bio-molecular interactions during the assay. The fluorescence mode was arranged to work at a different wavelength, since it is based on resonant excitation of a label close to the 1DPC surface. As shown in Figure 2c, it is possible to deliver the maximum energy at the surface when working at the resonant angle θ_2_ (λ_2_). In this particular condition, the dye can be resonantly excited in the absorption band (λ_2_). In the presence of the 1DPC, the excited molecules will emit preferentially into the radiative channels provided by the Bloch modes (TE mode). Hence the emission is enhanced and directed into the substrate through the 1DPC towards the detector [23]. The Alexa Fluor 647 was selected in order to have the emission spectrum close to λ_1_, corresponding to the angular emission peak in the window of the label-free operation.

The fabrication of the 1DPC was carried out by plasma ion assisted evaporation of dielectric materials (PIAD) under high vacuum conditions on standard microscope slides (Menzel). We used SiO_2_ (silica), Ta_2_O_5_ (tantala) and TiO_2_ (titania) as materials for the dielectric multilayers. The geometry of the 1DPC is sketched in Figure 2a. A first silica layer of 275 nm was used to provide defined conditions for the deposition of the multilayer on the glass substrate. The 1DPC consists of a periodic part made out of two tantala/silica (d_Ta_2_O_5__ = 120 nm, d_SiO_2__ = 75 nm) bilayers with a topping thin bilayer made out of titania/silica (d_TiO_2__ = d_SiO_2__ = 20 nm). The complex refractive indices of the dielectric materials were determined either by reflection/transmission spectroscopy on single supported thin films or by ellipsometry on test multilayers sustaining BSW to be n_SiO_2__ = 1.474 + i × 5 × 10^−6^, n_Ta_2_O_5__ = 2.160 + i × 5 × 10^−5^, and n_TiO_2__ = 2.28 + i × 1.8 × 10^−3^ at λ_1_ = 670 nm [12].

### 2.3. Optical Read-out System

Before its use, the bio-conjugated BSW biochip surface is topped with a fluidic cell and the back of the substrate is coupled to a BK7 glass prism by means of a refractive index matching oil. 

**Figure 3:**
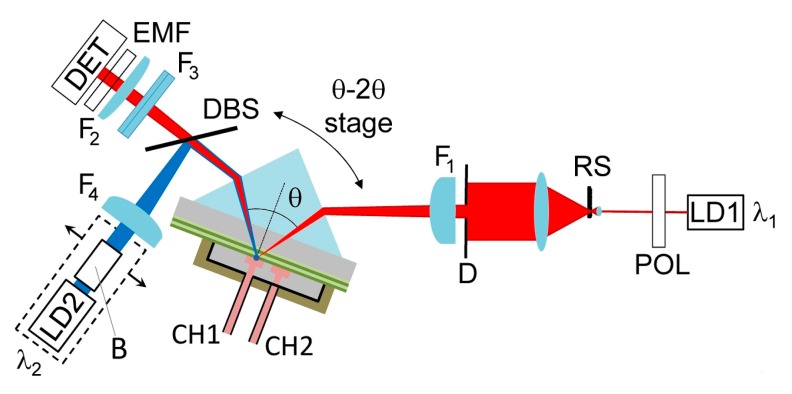
Simplified layouts of the optical setup used to interrogate the BSW chips in label-free and fluorescence operation modes.

**Label-free configuration**-Figure 3 shows the scheme of the optical read out system for the label-free operation (LF mode). A temperature stabilized (±0.01 °C) laser diode (LD1) at λ_1_ (Thorlabs LPS-675-FC) is collimated and TE polarized by means of a first polarizer (POL). The beam is then expanded by a telescope and subsequently focused by a cylindrical lens (F_1_ = 100 mm) into the coupling prism. A rotation stage is used to set the average incidence angle at θ_1_ and the angle of the detection arm at 2θ_1_. The reflected beam is imaged by a cylindrical Fourier lens (F_2_ = 75 mm) onto a monochrome CCD camera (Apogee Ascent, Sony ICX814 chip, W = 3388 pixel, H = 2712 pixel, corresponding to a sensor size of W = 12.50 mm and H = 10.00 mm, respectively). The long dimension W of the CCD is used to image the angular reflectance and achieve best sampling of the BSW resonance, with a 2.7° field of view, which is determined by W and f_2_. The conversion factor between pixels and angle is 7.9 × 10^−4^ deg/pix. The spots along the microfluidic channel are imaged along the short dimension H of the CCD by means of a properly positioned cylindrical lens (F_3_ = 150 mm). A rotating scatterer (RS), placed inside the telescope in the illumination arm, destroys the spatial coherence of the illumination beam and the CCD integration time is set to integrate the scattered light, thus ruling out speckle effects.

**Fluorescence configuration**-The fluorescence operation (FLUO mode) makes use of the same collection optics and CCD sensor (DET) used for the LF mode [23]. A polarized laser diode emitting at λ_2_ (LD2) is collimated and focused by a cylindrical lens (F_4_ = 130 mm) to a strip on the chip surface. A dichroic beam splitter (DBS, Chroma ZT 640 RDC) is used to reflect the excitation beam and transmits fluorescence emission. The collimation optics, the excitation filter (EXF, Chroma ZET 635/20) and the half wave plate (HWP) used to set the polarization to TE are positioned inside the excitation arm (B). The average incidence angle (θ_2_) can be tuned by translating the whole fluorescence excitation LD2 module (dashed box in tabure 3). An emission filter (EMF, Chroma 655 LP ET Longpass Filter) in front of the CCD cuts stray light from the excitation beam; such a filter transmits at λ_1_, therefore preserving the label-free operation.

### 2.4. BSW Chip Functionalization

The BSW chips functionalization procedure starts with a piranha cleaning solution (3:1 mixture of sulfuric acid and 30% hydrogen peroxide) for 10 min. This procedure permits removal of all organic contaminants from the sensitive surface and exposes the hydroxyl groups needed for the next functionalization step. The chips are then rinsed thoroughly with de-ionized (DI) water and dried under a stream of air. The chips are then immersed into a 2% solution of (3-aminopropyl) triethoxysilane APTES in ethanol/water (95:5 *v*/*v*) mixture at room temperature (RT) for 1h and, after removing from the APTES solution, are sonicated, rinsed with ethanol and baked on a hot plate at 110 °C for 1 h. In order to activate the primary amines of the APTES thin film, the chips are allowed to react with 1% (*v*/*v*) glutaraldehyde in 100 mM sodium bicarbonate buffer (pH 8.5) in the presence of 0.1 mM sodium cyanoborohydride for 1 h at ambient temperature (AT). Further sonication and rinsing in DI water follows. The glutaraldehyde-activated surface is then divided into two regions, the reference and signal region, by means of a hydrophobic marker that serves as a barrier during the bio-conjugation avoiding mixing. Such approach permits one to obtain a differential sensogram and to rule out all parasitic contributions due to local change of the temperature, pressure variations and non-specific interactions [12]. We verified that GAH-activated sensors can be stored for at least four weeks in low vacuum conditions without degradation. Once the bioconjugation is applied the biochips can be stored dry at +4 °C for a few days without any loss of the proteins activity.

### 2.5. Microfluidics

The microfluidic cell is composed of a microscope glass slide with four connection holes and a structured adhesive spacer (Lohmann Adhesive Tape GL-187, thickness 200 μm) to the two channels CH1 and CH2. By shifting the coupling prism in the read-out system, one can use either CH1 or CH2. The two parallel parts of the channels are 18 mm long, 1 mm wide and 2 mm distant from each other. The surface and volume of each channel are 63.5 mm^2^ and 12.7 μL, respectively. The fabrication of the cell was described elsewhere [14]. After gluing the microfluidic cell to the bio-conjugated 1DPC surface, the resulting BSW chip is topped by an aluminium back plate with a PDMS contact layer that provides the fluidic connections. The plate can be temperature controlled by means of a resistor and a Peltier element and assures a stable temperature of the biochip (±0.01 °C). During the experiments, the temperature was kept constant at T = 30 °C. The fluid handling system consists of a motorized syringe pump (1 mL, Cavro Centris Pump) and of a 10 positions stream selector (VICI Valco) allowing to select solutions from several different cuvettes. The connections were made out of PEEK tubings (250 μm inner diameter). In the assays, the injection of the solutions was performed at the flow rate 1.35 μL/s. During the injection steps, we adopted a recirculation procedure by pumping back and forth 40 times a volume 20 μL of the solution at a flow rate 1.35 μL/s.

## 3. Results and Discussion

In the following pages, we present some representative experiments showing the potential of BSW chips in revealing the ERBB2 in cell lysates at low concentration. First, we provide an insight on the on-chip and chip-to-chip variability as an example of a repeatable and reproducible cancer biomarker assay. Then, we describe an exemplary label-free sandwich assay making use of a PtG orienting layer to improve the bio-recognition efficiency of the capture mAb in presence of a complex biological matrix. In this approach, a detection mAb is also used to further amplify the mass load signal and increase assay selectivity. Finally, we describe an exemplary fluorescence sandwich assay, making use of a distinct capture mAb immobilization strategy and of a labeled mAb for detection. It is worth noting that the final achieved LoD depends on the optical detection scheme and, most importantly, on the bio-conjugation chemistry adopted to immobilize the capturing mAbs. The label-free sensitivity (S_V_ = 31.8 deg/RIU) of the BSW chips was determined experimentally by calibration tests carried out with glucose solutions in DI water at several different known concentrations [11].

### 3.1. On-Chip and Chip-to-Chip Repeatability

**Figure 4:**
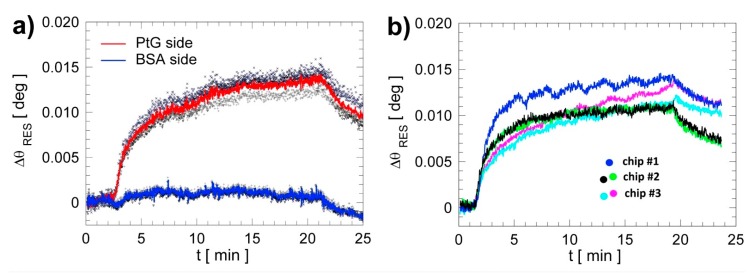
(**a**) On-chip variability on three spots in the signal and reference regions, solid lines correspond to averaged curves from signal (red) and reference (blue), respectively; (**b**) Chip-to-chip variability considering three different chips exposed to the same conditions (chemical functionalization and bio-conjugation protocols, assay characteristics and capture concentrations).

As shown in Figure 4, starting from BSW chips that were chemically functionalized according to the procedures described above, a solution of PtG (c = 0.5 mg/mL) was incubated on the signal region (PtG side) in order to produce a dense protein layer, which can efficiently bind and orient the capture mAb (Anti-ERBB2). As a control, we immobilized a BSA solution (c = 10 mg/mL) in the reference region (BSA side). In both cases the solutions were in D-PBS 1 X and were incubated for 1 h at AT. Subsequently, the chip was immersed in a BSA solution (10 mg/mL) in D-PBS 1 X to block the remaining reactive sites (overnight at 4 °C). Immediately before use, the surface of the BSW chips was always treated with a regeneration solution made of glycine in DI water and HCl (Gly-HCl, 10 mM, pH 2.0) for 2 min at RT. This procedure removes any adlayers formed on both the signal and reference regions upon BSA blocking.

To check repeatability of the bio-conjugation steps and of the LF assays, we performed several ERBB2 detection assays under the same conditions and focused our attention on the PtG/Anti-ERBB2 interaction. The results are shown in Figure 4. After recording a stable baseline in D-PBS 1 X, a solution of Anti-ERBB2 in D-PBS 1 X (volume 180 μL, 20 μg/mL) was injected in the chip. After 20 min we washed away the antibody solution coming back with D-PBS 1 X. This procedure was repeated with different pristine BSW chips and also with the same BSW chip, after regeneration with a solution of glycine (10 mM) and hydrogen chloride (Gly-HCl 10 mM, pH 2.0, see Table 1).

In Figure 4a, we show the sensograms recorded in three different spots in either the signal or the reference region of the same BSW chip: the spots in the signal region show a clear binding reaction of Anti-ERBB2 to the PtG layer; in the reference region no appreciable reaction takes place, as demonstrated by the almost flat behavior. The solid lines are the averaged signal (red) and reference (blue) curves, respectively. Figure 4a shows an excellent on-chip repeatability in terms of residual angular shifts. From the value of the residual angular shift after washing, (9.5 ± 0.9) mdeg, one can obtain the surface mass density of capture antibodies bound to PtG, (18.3 ± 1.7) ng/cm^2^ [12]. The maximum angular dispersion around the average value (between the three curves corresponding to the selected three spots) in the signal region is about 7% (see Table 1), indicating that the overall protocol is suitable to produce BSW chips with a controlled capturing surface density.

In Figure 4b, we show the average sensograms recorded in the signal regions of three different BSW chips (blue, cyan and black curves). As in the former case, we addressed the issue of repeatability in terms of the chip-to-chip variability of the Anti-ERBB2 differential residual signals (see Table 1).

From the results shown in Figure 3b one can evaluate the repeatability of the angular resonance shifts for different chips coming from the same deposition run but from different bio-conjugation runs. We find a maximum dispersion of 12%, demonstrating a good chip-to-chip repeatability.

The last case listed in Table 1 refers to chip-to-chip repeatability when considering also assays carried out with regenerated chips. The sensograms resulting from such assays are shown in Figure 4b too and were obtained with chip #2 (green) and chip #3 (purple) after a complete ERBB2 assay and a regeneration step with Gly-HCl. Including these assays in the analysis, the dispersion of the chip-to-chip average angular shift does not exceed 9%. In summary, on-chip and chip-to-chip variability remain in the low percent bracket, and the former is slightly better. This is expected, because the handling steps of separate chips are subjected to a greater experimental variability.

In terms of the reusability, we verified that PtG biochips can be reused, by repeating the regeneration step, up to three times without unmounting them from the optical set-up. In addition, we verified that the BSW biochips can be reused, after piranha cleaning (biochips unmounted from the platform) up to 15 times before delamination of the 1DPC can occur.

### 3.2. Label-Free Cancer Biomarker Detection Assay

BSW chips prepared according to the procedures described in the above Section 3.1 were used to perform label-free cancer biomarker detection. In order to incorporate further referencing, a control lysate (Colo 38) was also used in the assays. The protocol is divided in three steps: Anti-ERBB2 capture antibody injection (compare Section 3.1), lysate interaction, Anti-ERBB2 antibody amplification. In-between these steps a washing routine in D-PBS 1 X was performed.

To detect ERBB2 molecules in SK-BR 3 and Colo 38 lysates, we developed an ad hoc cancer biomarker assay. Since two fluidic channels are available for sensing, the same BSW chip can be used to sense the ERBB2 positive and negative lysates sequentially due to the particular optical configuration of the illumination beam. In order to guarantee the same experimental conditions in both microfluidic channels, we set identical washing steps, reaction times, flow rates, anti-ERBB2 mAbs, and whole lysate concentrations.

**Figure 5:**
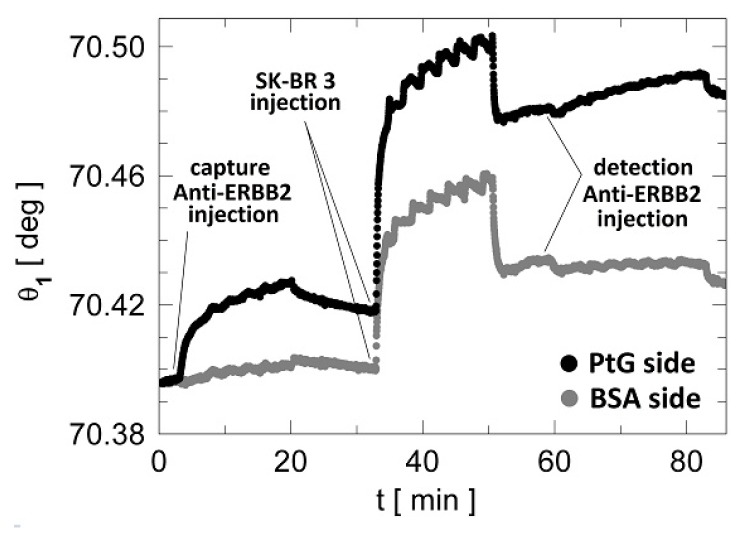
Resonance minimum position as a function of time during a complete label-free cancer biomarker bio-recognition assay. The curves are averaged on three adjacent spots and correspond to the PtG signal (black) and BSA reference (grey) regions, respectively. Note that the duration of a complete assay is about 80 min.

In Figure 5, we show the average (three spots) sensograms recorded in both the signal and the reference regions during an ERBB2 detection assay in a lysate from SK-BR 3 cells. The initial washing step involves a 500 μL injection of D-PBS 1 X to get a stable baseline of the sensogram. Once a stable signal is reached, an injection of the Anti-ERBB2 capture antibody (180 μL, 100 μg/mL) is performed to confer selectivity only to the signal region exploiting the PtG/Anti-ERBB2 interaction (Figure 5, between 3 and 20 min). After a washing step in D-PBS 1 X, either SK-BR 3 or Colo 38 cell lysates were injected (180 μL of NP40 1% extracts) in either the CH1 or the CH2. The whole lysate concentrations were D0 = 1.3 μg/mL for the ERBB2 positive and negative lysates, corresponding to an estimated ERBB2 concentration in the SK-BR 3 sample of 0.5 ng/mL. During lysate injection, it was clearly observed that the amount of NP40 used for cells lysis influenced the injection procedures in the channels, most likely because detergents generate air bubbles and micelles. This is expected to cause a degradation in the label-free signal (see the signal in the Figure 5 at times in the 32–52 min range). Consequently, the lysate phase could hardly be interpreted in the presence of large oscillations largely due to the recirculation effects and to the presence of the non-ionic detergent in the samples. Moreover, the residual angular shift recorded after lysate interaction could not be unequivocally ascribed to ERBB2 specific interactions. Yet, a specific recognition of antigens in the lysate was possible thanks to the second detection event mediated by the soluble anti-ERBB2 mAb. After a washing step in D-PBS 1 X, a final injection of the detection Anti-ERBB2 (180 μL, 17 μg/mL) was performed (58–82 min). This step yields additional selectivity as well as an amplification of the label-free signal, thus producing in the case of SK-BR 3 a supplementary angular shift.

**Figure 6:**
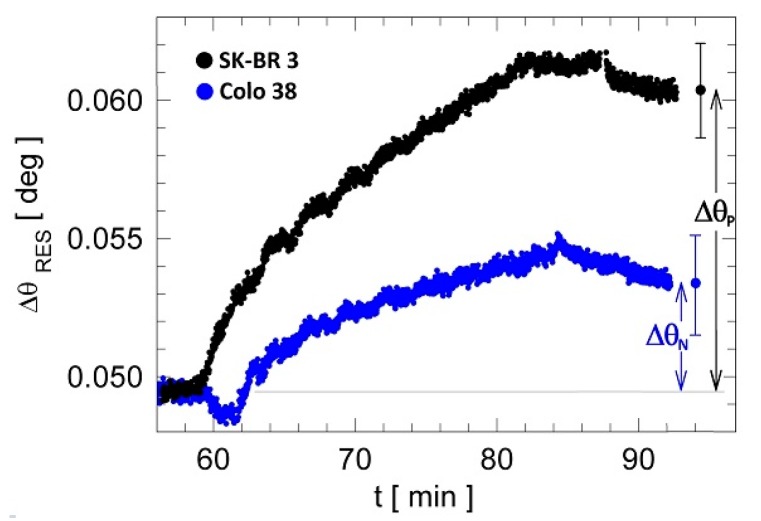
Differential signals for SK-BR 3 and Colo 38 lysates during the detection phase (Anti-ERBB2 interaction). The standard deviations of the mean for the differential sensograms and the residual angular shifts are also shown: Δθ_P_ for SK-BR 3 and Δθ_N_ for Colo 38.

Next, the assays were repeated under the same conditions for the control lysate (Colo 38), and a sensorgram was elaborated by subtracting the average curves in the signal and reference regions (Figure 6). Anti-ERBB2 detected differential residual angular shifts (Δθ_P_ and Δθ_N_) in SK-BR 3 and Colo 38 lysates, respectively; the error bars indicate the uncertainties σ_SK-BR 3_ = 0.0034° and σ_Colo 38_ = 0.0036°, which were calculated as the standard deviation of the mean of the difference signal. The lysates’ interaction results in a net difference between the SK-BR 3 and Colo 38 residual signal Δθ_P_ − Δθ_N_ = 0.0073°, which is larger than 2σ_SK-BR 3_, therefore demonstrating that ERBB2 is detectable with the presented approach at least at 0.5 ng/mL. Therefore, we can assume that in the label-free mode LoD_LF_ = (0.5 ± 0.1) ng/mL, which corresponds to (2.5 ± 0.5) pM. From the analysis of the signals and uncertainties shown in Figure 6 and performing a linear extrapolation, which is likely given the small concentration span, we find that such LoD_LF_ could be pushed a bit down to 0.4 ng/mL.

The Colo 38 residual angular shift could be due to a basal expression of ERBB2 molecules for such a cell lysate, which, however, cannot be estimated under these experimental conditions since Δθ_N_ is anyhow within 2σ_Colo 38_. Such result suggests that the resolution for ERBB2 detection could be improved, in case a better negative control is used.

Altogether, these results confirm the quality of the newly developed assay for cancer biomarker detection and pave the way to obtain an ultimate limit of detection in label-free mode that may be in the range of state-of-art techniques presently used in cell biology.

### 3.3. Fluorescence Cancer Biomarker Detection Assay

The BSW chips used in the exemplary assays carried out in the fluorescence mode were prepared according to a different protocol. The capture Anti-ERBB2 (1 mg/mL) and a reference Anti-MHC I (1 mg/mL) were incubated and immobilized directly onto the glutaraldehyde-activated surface for 1 h at AT in the signal and reference regions, respectively. At the end of the immobilization procedure, the chips were immersed in a solution of BSA (10 mg/mL) in D-PBS 1 X to block the remaining reactive sites (overnight at 4 °C). Immediately before their use in a detection assay, the surface of the BSW chips was always treated with with Gly-HCl for 2 min at AT. With respect to the label-free detection assays reported above, the absence of the PtG layer and the removal of the injection of the capturing mAb on a PtG activated BSW chip permit reducing the assay duration (45 min) but also drop the orientation step of the capturing Anti-ERBB2. When using statically oriented mAbs on activated-GAH surfaces, the capturing performances of the surface were reduced with respect to the PtG case (Figure S1, Supplementary Materials). Nevertheless, due to the non-covalent nature of the PtG-mAb interaction and the possible non-specific recognition of the detection mAbs, we decided to implement the assay grafting the capture mAb directly on the GAH-sensitive surface.

In this format, we diluted the SK-BR 3 lysate with lysis buffer to obtain different ERBB2 concentrations. The whole protein content c_WH_ in the resulting four dilutions (D4 to D1) was: 500 μg/mL (D4, C_ERBB2_ = 100 ng/mL), 200 μg/mL (D3, C_ERBB2_ = 50 ng/mL), 50 μg/mL (D2, C_ERBB2_ = 10 ng/mL) and 10 μg/mL (D1, C_ERBB2_ = 2 ng/mL). In the assay, after a washing step in D-PBS 1 X, known dilutions of SK-BR 3 lysates were injected into a freshly prepared BSW chip. After a 20 min incubation, the flow channel was washed with D-PBS 1 X. Then, the labelled detector (Anti-ERBB2 AF 647; same mAb used in label-free measurements) was injected and incubated for 20 min. After a final washing step in D-PBS 1 X, we performed the fluorescence measurement. The fluorescence excitation LD2 module is translated to excite the chip at the resonant BSW angle θ_2_ at the excitation wavelength λ_2_ and the AF 647 emission around λ_1_ is collected by the CCD through the detection optics. Before starting any assay, the fluorescence background was recorded before labelling and subtracted from the fluorescence signals for normalization.

**Figure 7:**
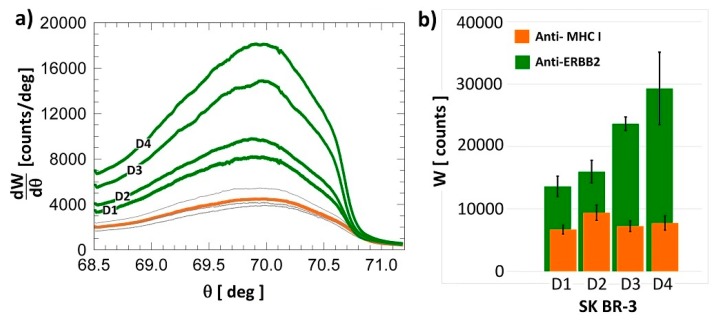
(**a**) Fluorescence intensity signals for four different SK-BR 3 lysate dilutions (green curves from D1 to D4). The orange curve is the average fluorescence intensity for the reference spots in four different BSW chips (light grey curves); (**b**) Corresponding integrated fluorescence intensity in the signal region (Anti-ERBB2, green bars) and in the reference region (Anti-MHC I, orange bars).

Figure 7a shows the background subtracted fluorescence intensities recorded in the signal region for the four SK-BR 3 diluted samples (green curves). Each curve was obtained with a freshly prepared BSW chip. The intensities are proportional to the ERBB2 content in the samples, as expected. Such spectra can be compared to the signals collected from the reference region where the control mAb (Anti-MHC I) was immobilized (grey curves in Figure 7a) for the four chips. In Figure 7b, the integrated intensity is plotted for the four dilutions in the signal W_SIG_ and reference spots W_REF_.

**Figure 8:**
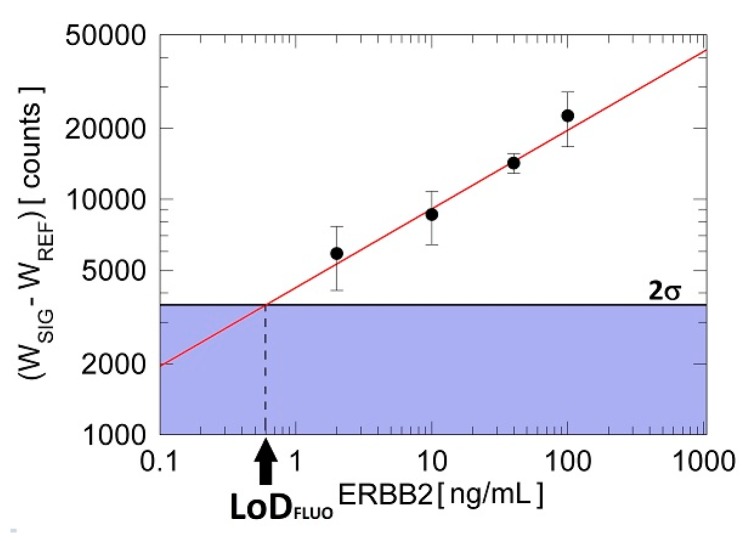
Reference subtracted integrated fluorescence intensities as a function of ERBB2 concentrations (from D1 to D4). The limit of detection (2σ) is determined from the standard deviation of the smallest concentration tested in the experiments.

In Figure 8, the measured differential integrated fluorescence intensities W_SIG_-W_REF_ are plotted as a function of the amount of ERBB2 in the SK-BR 3 lysate dilutions is shown. As it may be seen, the red fitting line confirms that W_SIG_-W_REF_ is linear in a logarithmic scale in the ERBB2 concentration range explored. The noise level was set at 2σ, where σ is the standard deviation of the mean of the differential fluorescence signal obtained for the smallest ERBB2 concentration measured. In the fluorescence operation mode, ERBB2 could be clearly detected at concentrations as low as 2 ng/mL.

Thus, from Figure 8 we can evaluate the limit of detection of the platform when operated in the fluorescence mode (LoD_FLUO_) for the ERBB2 cancer biomarker. LoD_FLUO_ is the concentration at which the fit reaches 2σ [24,25]. Our estimated LoD_FLUO_ = (0.6 ± 0.1) ng/mL corresponds to (3.2 ± 0.5) pM. Of course, an analytical quantification of such a biomarker in cell lysates should be performed after a calibration with solutions of known concentration of ERBB2. However, at the present stage, such a calibration curve is being constructed but is not yet reported in the present work.

As pointed out in the introductory section, our BSW chips can be used as a valid alternative to standard techniques, such as WB. In Figure S2 we show a WB assay performed with the same SK-BR 3 cell lysates measured with the presented platform. It is clear that the lysate concentrations D0 and D1 (the smallest detected with our technique) cannot be revealed by means of a standard WB. Moreover, a comprehensive comparative table can be found in [8] showing the performances of state of the art point-of-care ERBB2 detection platforms. According to such a table, the presented approach has one of the lowest LoD and the shortest assay time (45 min).

## 4. Conclusions

In this work, we presented BSW chips making use of 1DPC as optical transducer elements for cancer biomarker detection. Specifically, we described the use of BSW chips in two modes: label-free and fluorescence. After studying the on-chip and chip-to-chip variability of the detection antibody binding step (that was in the low percent range), we focused our attention on the optimization of a cancer biomarker assay for ERBB2 positive and control lysates. For the label-free case, we studied the use of PtG in a cancer biomarker assay and the benefits introduced from such orienting protein reaching the ultimate LoD_LF_ of 0.5 ng/mL. In addition, we demonstrated that BSW chips can also be used in fluorescence operation mode. To this end, we selected a series of SK-BR 3 dilutions to evaluate the amount of ERBB2 in cell lysates. The LoD_FLUO_ attained in these particular conditions is 0.6 ng/mL, positioning both techniques in the ELISA concentration range. It is important to note that the LoD_LF_ was obtained with the orienting PtG layer and without fluorescently labelling the detection Anti-ERBB2 antibody. It is well known that the labeling procedure can deteriorate the binding capacity of mAbs reducing their efficiency when used in specific bio-recognition assays. On the other hand, in fluorescence mode, we considerably reduced the total assay time to only 45 min. Finally, the LoDs obtained in label-free and fluorescence modes are well below the threshold (15 ng/mL) set from the US Food and Drug Administration in 2000, confirming the BSW chips as a valid alternative to conventional techniques for the detection of soluble cancer biomarkers, such as ELISA.

## Figures and Tables

**Table 1:** Values of the characteristic parameters extracted from Figure 3.

	Average Δθ (mdeg)	Γ (ng/cm^2^)	Conditions
on-chip	(9.7 ± 0.7)	(18.6 ± 1.3)	3 spots on the same chip
chip-to-chip	(10 ± 1.2)	(19.2 ± 2.3)	3 different chips
chip-to-chip ^1^	(9.5 ± 0.9)	(18.3 ± 1.7)	3 different chips after regeneration

^1^ after exposing to chips to a regeneration solution.

## Supplementary Materials

The following are available online at www.mdpi.com/2079-6374/7/3/33/s1, Figure S1: Injection of the detection mAb Anti-ERBB2 on a BSW chip prepared with statistically oriented capture mAb covalently bound to a GAH-activated surface and exposed to a SK-BR 3 cell lysate. The sensograms for signal (blue curve) and reference (black) regions are reported on the left axis (expressed in angular camera pixel). The red curve is the differential signal expressed in angle displacement after proper conversion (Section 2.3 in the main manuscript), Figure S2: Western blot analysis of ERRB2 in SK-BR 3 cell lysate at different concentrations (μg/lane). The D0, D1, D2 and D3 are the SK-BR 3 concentrations experimentally measured with our BSW chips in both label-free and fluorescence modes.
