# Supplementary material for: Bloch Surface Waves Biosensors for High Sensitivity Detection of Soluble ERBB2 in a Complex Biological Environment
Source: Biosensors (Basel). 2017 Aug 17;7(3):33. doi: 10.3390/bios7030033 (PMC5618039; doi:10.3390/bios7030033)
Supplement: Supplementary file 1 [file biosensors-07-00033-s001.pdf]

Supplementary Materials: Bloch surface waves biosensors for high sensitivity detection of soluble ERBB2 in a complex biological environment

Alberto Sinibaldi, Camilla Sampaoli, Norbert Danz, Peter Munzert, Frank Sonntag, Fabio Centola, Agostino Occhicone, Elisa Tremante, Patrizio Giacomini, and Francesco Michelotti

This figure shows the injection of the detection mAb (det mAb Anti-ERBB2) for a SK-BR 3 assay when the BSW chip is prepared by means statically oriented capturing mAbs on activated-GAH surface. Even if, in this case, the concentration of the capture mAb is larger (1 mg/mL), we can record a smaller differential angular shift ($\Delta\theta_{PtG\text{-}free}$ = 6.7 mdeg, red curve) with respect to PtG-based BSW chips ($\Delta\theta_{PtG}$ = 11 mdeg, Fig.6 in the main manuscript). The SK-BR 3 assay conditions were comparable with the PtG-based BSW chips.

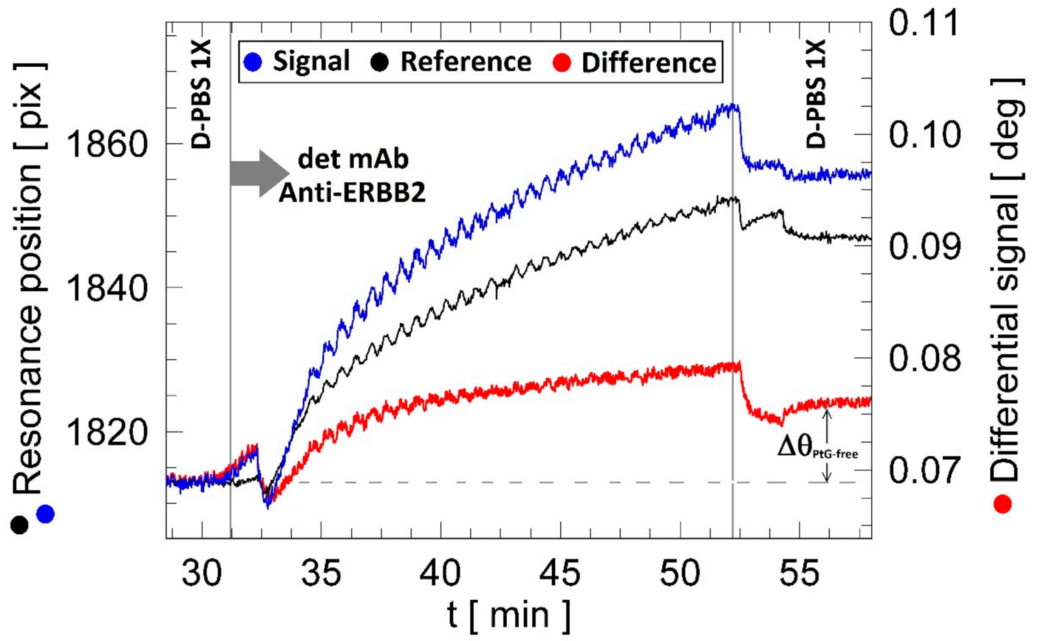

Figure S1: Injection of the detection mAb Anti-ERBB2 on a BSW chip prepared with statistically oriented capture mAb covalently bound to a GAH-activated surface and exposed to a SK-BR 3 cell lysate. The sensograms for signal (blue curve) and reference (black) regions are reported on the left axis (expressed in angular camera pixel). The red curve is the differential signal expressed in angle displacement after proper conversion (Section 2.3 in the main manuscript).

In Fig.2S we provide a comparison of the results obtained on our platform with independent WB experiments. In order to compare the results, the concentrations of SK-BR 3 cell lysates loaded per lane were identical with the ones measured with our BSW chips. As it can be seen, the smallest concentrations revealed in label-free (D0) and fluorescence (D1) modes were not detectable with a standard WB technique. This verifies the effectiveness of our development as compared to a well established technique as WB, assessing the advantage and uniqueness of our development.

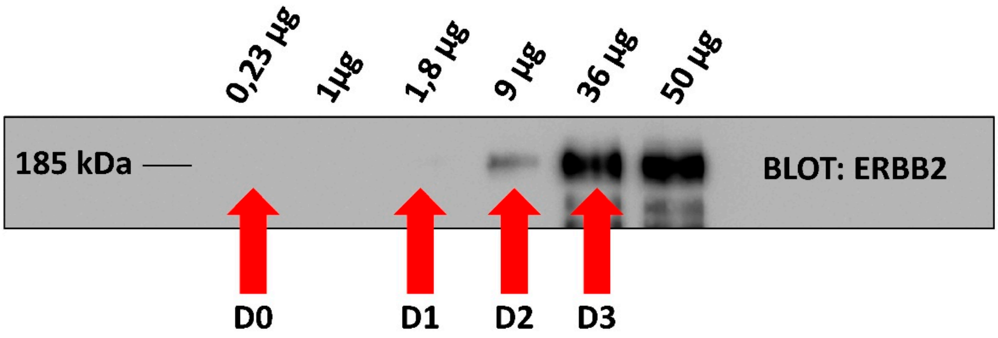

Figure S2: Western blot analysis of ERRB2 in SK-BR 3 cell lysate at different concentrations (µg/lane). The D0, D1, D2 and D3 are the SK-BR 3 concentrations experimentally measured with our BSW chips in both label-free and fluorescence modes.
